# The Expression of the Alpha7 Nicotinic Acetylcholine Receptor and the Effect of Smoking in Curdlan-Administered SKG Mice

**DOI:** 10.3390/biomedicines11102757

**Published:** 2023-10-11

**Authors:** Young-Eun Kim, Jae-Hyun Lee, Eun-Ju Lee, Do Hoon Kim, Mi Ryeong Jeong, Seokchan Hong, Chang-Keun Lee, Bin Yoo, Jeehee Youn, Eun-Ju Chang, Yong-Gil Kim

**Affiliations:** 1 Department of Rheumatology, Asan Medical Center, University of Ulsan College of Medicine, Seoul 05505, Republic of Korea; tph00066@naver.com (Y.-E.K.); clesote@hanmail.net (J.-H.L.); krys72@hanmail.net (E.-J.L.); alakzam41@naver.com (D.H.K.); miryeong21@gmail.com (M.R.J.); hongsc97@hanmail.net (S.H.); cklee@amc.seoul.kr (C.-K.L.); byoo@amc.seoul.kr (B.Y.); 2 Department of Anatomy and Cell Biology, College of Medicine, Hanyang University, Seoul 04763, Republic of Korea; jhyoun@hanyang.ac.kr; 3 Department of Biomedical Sciences, Asan Medical Center, University of Ulsan College of Medicine, Seoul 05505, Republic of Korea; ejchang@amc.seoul.kr

**Keywords:** alpha7 nicotinic acetylcholine receptor, smoking, nicotine, spondyloarthropathy, interleukin-17, arthritis

## Abstract

Nicotine, an abundant molecule in tobacco, has immunomodulatory effects on inflammatory diseases, primarily due to the activation of alpha7 nicotinic acetylcholine receptor (α7 nAChR). We aim to evaluate the expression of the α7 nAChR^+^ cells in joint tissue and the effect of smoking on immune cells and peripheral arthritis in curdlan-administered SKG mice, a murine model of spondyloarthropathy (SpA). The SKG mice were injected with curdlan two times at 2-week intervals and were divided into two groups; one exposed to cigarette smoke and the other not exposed. We found that the α7 nAChR^+^ cells increased in the joint tissue of curdlan-administered SKG mice compared to in the wild type. Furthermore, the peripheral arthritis scores and histological scores for synovial inflammation were lower in smoke-exposed curdlan-administered SKG mice than in mice not exposed to smoke. Immunofluorescence staining of the α7 nAChR^+^ and IL-17A^+^ cells was lower in the synovia of smoke-exposed mice than the control mice. The proportions of α7 nAChR^+^IL-17A^+^ and α7 nAChR^+^IL-17A^+^FOXP3^+^ cells also decreased in the synovia of smoke-exposed mice compared with the controls. We observed an increase in the α7 nAChR^+^ cells within the joint tissue of curdlan-administered SKG mice and that cigarette smoke had an influence on both peripheral arthritis and immune cell population, especially α7 nAChR^+^ cells. Thus, exposure to cigarette smoke after arthritogenic stimuli may have an anti-arthritogenic effect in curdlan-administered SKG mice.

## 1. Introduction

Spondyloarthropathy (SpA) is an inflammatory rheumatic disease characterized by chronic back pain, arthritis, enthesitis, and dactylitis [1]. Recently, interleukin-17 (IL-17)-producing cells, especially T helper 17 (TH17) cells, have been found to play pro-inflammatory roles in the pathogenesis of SpA [2,3]. Cytokines including IL-23 and TGF-β induce TH17 differentiation, and the TH17 cells generate IL-17, activating osteoclasts and inhibiting bone regeneration [4]. Serum IL-17 levels are also higher in ankylosing spondylitis (AS) patients than healthy controls [5], and the increase in the number of TH17 cells in patients with AS may be due not only to the preferential differentiation of naive T cells but also to a reduction in the plasticity of the TH17 cells [6]. Interestingly, Forkhead box P3^+^ cells (FOXP3^+^), which are immunosuppressive cells that maintain immune homeostasis, were found to undergo conversion into TH17 cells in the synovia of rheumatoid arthritis (RA) patients and arthritic DBA1 mice, pointing to a transition of FOXP3^+^ T cells into TH17 cells [7,8].

Smoking is a poor prognostic factor for RA and AS. Smokers have higher levels of rheumatoid factor (RF) and are likely to develop RA [9,10]. However, collagen-induced arthritic (CIA) mice exposed to smoking had lower levels of anti-cyclic citrullinated peptide (anti-CCP), as well as a delayed onset and progression of arthritis [11]. It is thought that the anti-inflammatory effects of smoking are caused by nicotine’s activation of the cholinergic anti-inflammatory pathway. The cholinergic anti-inflammatory pathway, which is implicated in interactions between the autonomic nervous system and the immune system, may also play a role in autoimmune arthritis by regulating cytokine levels and the activities of immune cells [12]. Alpha7 nicotinic acetylcholine receptor (α7 nAChR), expressed in a variety of immune cells, including TH17 cells, is a component of cholinergic pathways; it not only inhibits the release of cytokines but also regulates the activity of immune cells [12]. Treatment of fibroblast-like synoviocytes (FLS) isolated from rheumatoid arthritis (RA) patients with agonists of α7 nAChR reduced the levels of the pro-inflammatory cytokines IL-6 and IL-8 [13]. Additionally, daily administration of α7 nAChR agonists via oral gavage or intraperitoneal injection into CIA mice reduced their clinical arthritis and histological scores [14,15]. Although previous research has shown that α7 nAChR agonists can ameliorate inflammatory arthritis, there have been few reports about the effect of cigarette smoke, which exerts the function of an α7 nAChR agonist on inflammatory arthritis, especially SpA [16].

This immunomodulatory effect of smoking is attributed to nicotine, despite its numerous detrimental effects on immune-mediated diseases [17,18]. Nicotine primarily acts via nAChRs on immune cells, initiating the cholinergic anti-inflammatory pathway, leading to the suppression of pro-inflammatory cytokines like TNF-α and IL-1β [19]. Oral administration of nicotine reduced the IL-17A levels in CIA mice by activating α7 nAChR on the TH17 cells and led to improved clinical arthritis scores [18,20]. In addition, intraperitoneal administration of nicotine to DBA1 mice reduced macrophage accumulation in synovia, accompanied by reduced monocyte chemoattractant protein (MCP)-1 and macrophage inflammatory protein (MIP)-1α, suggesting that it decreased macrophage migration [21]. Given the well-documented health risks associated with nicotine, cotinine emerges as a promising alternative immunomodulatory agent. Cotinine is a metabolite of nicotine [22] and downregulates inflammation by reducing the production of various pro-inflammatory cytokines using α7 nAChR [23] as an alternative immunomodulatory agent. Cotinine has a longer half-life than nicotine [24], and might be non-addictive and safe; therefore, its anti-inflammatory effects deserve study.

Considering the reported relationship between AS and smoking in epidemiologic studies [25], the effects of smoking on immune cells and arthritis in SpA are worth researching. However, CIA mice, a widely used autoimmune arthritis model, do not suffer from SpA, and self-resolution occurs around 60 days after immunization; IL-17A has a major impact on arthritis, but arthritis occurs when IL-17A is deficient [26]. SKG mice provide a better model of human SpA, as they display peripheral arthritis, enthesitis, and inflammatory bowel disease [27]. These mice have a point mutation in the SH2 domain of ZAP-70, a gene encoding a key signal transduction molecule in T cells [28].They have the potential to cause peripheral arthritis at an early stage and spinal ankylosis at a later stage, due to the development of autoreactive T cells, in part as a result of IL-17A/IL-22 and IL-23 signaling, and they are especially sensitive to beta-glucans such as curdlan [29,30]. Curdlan, a β-glucan polymer, is synthesized by various bacterial genera, notably Agrobacterium, Alcaligenes, and Rhizobium, and is able to stimulate Toll-like receptor 2 (TLR2) signaling and, consequently, to induce a Th17 cell-mediated immune response. In our study, curdlan serves as a potent arthritis-inducing agent in SKG mice [27].

In the present study, we aimed to evaluate the level of α7 nAChR expression in the SKG model of autoimmune arthritis and assess the effects of exposure to cigarette smoke on peripheral arthritis and spinal ankylosis following the administration of curdlan.

## 2. Materials and Methods

### 2.1. Induction of Arthritis and Ankylosis in SKG Mice

The SKG mice were obtained from Dr. S. Sakaguchi [26]. Eight-week-old male SKG mice were intraperitoneally injected with either 3 mg of curdlan (FUJIFILM Wako Chemicals, Richmond, CA, USA) suspended in 0.2 mL of PBS (*n* = 34) or 0.2 mL of PBS without curdlan (*n* = 34) at 0 and 2 weeks. Control, non-model, 8-week-old male BALB/c mice were intraperitoneally injected with 0.2 mL of PBS at 0 and 2 weeks (*n* = 17). While female SKG mice exhibit severe arthritis in the early phase [28,31,32], male SKG mice were chosen to investigate the impact of smoke exposure more effectively on arthritis development. All mice were maintained in a specific pathogen-free facility under a 12 h light-to-dark cycle, with free access to food and water. 

### 2.2. Cigarette Smoke Exposure

Then, one-half of the curdlan-administered male SKG mice (*n* = 17) and the PBS-administered male SKG mice (*n* = 17) were exposed to smoke from 3R4F cigarettes (8.0 mg of tar/cigarette and 0.70 mg of nicotine/cigarette, Camel) as described in a previous study, with some modifications [33]. The group not exposed to cigarette smoke inhaled only room air. After gradual escalation to allow the mice to adapt to the smoke, they were exposed three times to smoke from four cigarettes for 15 min each time, with a 10 min rest in between. This exposure was repeated three times, 5 days a week for 20 weeks, starting at the time of the first curdlan injection. Briefly, all mice were placed in an inhalation box (50 × 40 × 30 cm) connected to a pump and exposed to mainstream cigarette smoke generated by burning four cigarettes simultaneously for 15 min. Then, the box was ventilated to remove the cigarette smoke, and the mice breathed normal room air for 10 min. This process was repeated for two additional exposures.

### 2.3. Liquid Chromatography-Tandem Mass Spectrometry (LC-MS/MS) for Metabolomic Analysis

At 16 weeks post curdlan injection, the mice’s blood was collected and metabolomic analysis for nicotine and cotinine was undertaken. The blood was allowed to clot for a minimum of 1 h at room temperature and centrifuged at 16,000× *g* for 15 min at 4 °C. The separated mouse serum was collected and stored at −80 °C. The cotinine standard and internal standard were purchased from Cayman Chemical Company. All solvents including water were purchased from J. T. Baker. 5 μL of the mouse serum was mixed with 500 uL of ethyl acetate and 50 μL of the internal standard solution (1 uM of the cotinine-d3 solution). The solution was sonicated for 30 min at room temperature at ultrasonic frequency (40 kHz) and then centrifuged at 13,000 rpm for 15 min at 4 °C. The supernatant was collected and dried using a vacuum centrifuge. The dried matter was stored at −20 °C, and reconstituted with 50 μL of acetonitrile prior to LC-MS/MS analysis. The cotinine was determined using an LC-MS/MS system equipped with 1290 HPLC (Agilent, Waldbronn, Germany) and QTRAP 5500 (ABSciex, Toronto, ON, Canada). A reverse-phase column (Pursuit5 C18, 150 × 2.1 mm) was used with mobile phase A (0.1% formic acid in H_2_O) and mobile phase B (0.1% formic acid in methanol). The LC run was performed with an isocratic condition of 90% B with 300 µL/min for 10 min, and the column temperature was kept at 30 °C. 10 μL was injected into the LC-MS/MS system and ionized with a TurbolonSpray ionization source. Multiple reaction monitoring (MRM) was performed in the positive ion mode and the extracted ion chromatogram corresponding to the specific transition (m/z 177.2 → 80.0) for cotinine was used for quantification. The calibration range for cotinine was 0.01–10 nM (r2 ≥ 0.99). Data analysis was performed using the Analyst 1.7.1 software (Sciex, Framingham, MA, USA).

### 2.4. Clinical Scoring of Peripheral Arthritis

The clinical peripheral arthritis scores of mice were measured every week for up to 21 weeks post curdlan injection on a scale of 0–4 (0 = no swelling, 1 = mild swelling and redness on the top of the foot, 2 = severe swelling and redness on the top of the foot, 3 = severe swelling and redness of the wrist or ankle joints, and 4 = severe swelling of the wrist or ankle joints and digits) [27]. The arthritic scores of the affected joints were combined to obtain a composite score for all paws.

### 2.5. Histological Analysis

All mice were sacrificed at 23 weeks post curdlan injection, and the left hind regions were collected. The excised left hind regions were fixed in 10% buffered formalin and incubated in Calci-Clear Rapid (National Diagnostics, Atlanta, GA, USA) for 48 h for decalcification. Fixed tissues were rinsed with tap water to remove the formalin for about 2 h. They were then dehydrated in graded ethanol, cleared with xylene using a tissue processor (Excelsior AS Shandon Diagnostics Ltd., Runcorn, UK), and embedded in paraffin using a paraffin embedding station (EG1150H; Leica, Wetzlar, Germany). Using a rotary microtome (RM2255; Leica, Wetzlar, Germany), the paraffin blocks were cut into 3 µm thick sections, which were placed onto glass slides. Hematoxylin and eosin (H&E) staining and mounting with coverslips were performed using an automatic stainer (Leica Autostainer XL, Wetzlar, Germany). Semiquantitative scoring of the joint inflammation severity on a scale of 0–3 was performed in accordance with standardized microscopic arthritis scoring of histological sections (SMASH) [34]. All animal experiments were performed in accordance with the guidelines for animal care approved by the Animal Experimentation Committee of the Asan Institute for Life Sciences (2021-14-226).

### 2.6. Immunofluorescence Staining

Multiplexed immunofluorescent staining of the mouse hind paws or spines was performed using the Opal method (Perkin Elmer, Waltham, MA, USA), but to avoid fluorophore spillover, we kept the exposure time for each channel as short as possible and ensured that the Opal fluorophores corresponded with a well-defined wavelength range that did not overlap with adjacent channels. The primary antibodies were applied sequentially to a single slide, as described below. The slides were deparaffinized with xylene and rehydrated using ethanol. For antigen retrieval, they were heated in a citrate buffer (pH 6.0) using a microwave. The slides were first incubated with primary rabbit antibodies to α7 nAChR (ab216485, 1:500; Abcam, Cambridge, MA, USA) in a humidified chamber at 37 °C for 1 h and then with horseradish peroxidase (HRP) Mouse (Ms) + Rabbit (Rb). The alpha7 nAChR was visualized using fluorescein Opal 520 (1:100). Then, the slides were placed in citrate buffer (pH 6.0) and heated using a microwave. After they had cooled, they were incubated with the primary rabbit antibody to FOXP3 (ab215206, 1:1000; Abcam, Cambridge, MA, USA) overnight (~14 h) in a humidified chamber at 4 °C, followed by detection using polymer HRP Ms + Rb. FOXP3 was visualized in a similar way using Opal 620 (1:100) and the primary rabbit antibody against IL-17A (NBP1-76337, 1:500; Novus Biologicals, Centennial, CO, USA). IL-17A was visualized using Opal 690 (1:100). Lastly, the slides were again placed in citrate buffer (pH 6.0) and heated in a microwave. The nuclei were visualized with DAPI (1:500), and the sections were mounted using a mounting medium (HIGHDEF^®^ ADI-950-260-0025; Enzo Life Sciences, Farmingdale, NY, USA). They were then incubated with rabbit antibodies against F4/80 (ab111101, 1:100; Abcam, Cambridge, MA, USA) as described above. For antigen retrieval, the slides were heated in a Tris-EDTA buffer (pH 9.0) using a microwave. The slides were then incubated with primary antibodies against F4/80 overnight in a humidified chamber at 4 °C. F4/80 was visualized using Opal 690 (1:100). The stained slides were scanned using SLIDEVIEW VS200 (Olympus, Tokyo, Japan). The data were analyzed using HALO v3.1.1076 (Indica Labs, Albuquerque, NM, USA).

### 2.7. Imaging with a Fluorescent In Vivo Bisphosphonate Agent

Whole-body imaging was performed at 21 weeks post curdlan injection after depilation of the back using the fluorescent in vivo bisphosphonate agent OsteoSense 680 EX (PerkinElmer, Waltham, MA, USA). The mice were injected with the bisphosphonate agent (2 nmol in 100 μL of PBS) via the tail vein. After 18 h, fluorescent images were obtained using an IVIS spectrum system (Perkin Elmer, Waltham, MA, USA) at excitation and emission wavelengths of 675 and 720 nm, respectively, under inhalation anesthesia. After acquisition, the images were spectrally unmixed (Living Image 4.2 software; Caliper Life Sciences, Hopkinton, MA, USA). ROIs of the same area were placed and the mean radiant efficiencies measured.

### 2.8. Statistical Analysis and Schematic Diagrams

All analyses were performed using the GraphPad Prism 8.4.3 software (GraphPad Software, San Diego, CA, USA). Mann–Whitney U tests were performed for two-group comparisons. We used ANOVA tests adjusted for multiple comparisons using Bonferroni correction for a comparison of more than two groups. *p* values less than 0.05 were considered statistically significant. The results are presented as means ± standard errors of the means (SEM). Significant differences are indicated with asterisks as follows: * *p* ≤ 0.05, ** *p* ≤ 0.01, *** *p* ≤ 0.001, and **** *p* ≤ 0.0001. All the schematic diagrams were created at BioRender.com.

## 3. Results

### 3.1. Expression of α7 nAChR in the Joint Tissue of Curdlan-Administered SKG Mice

**Figure 1:**
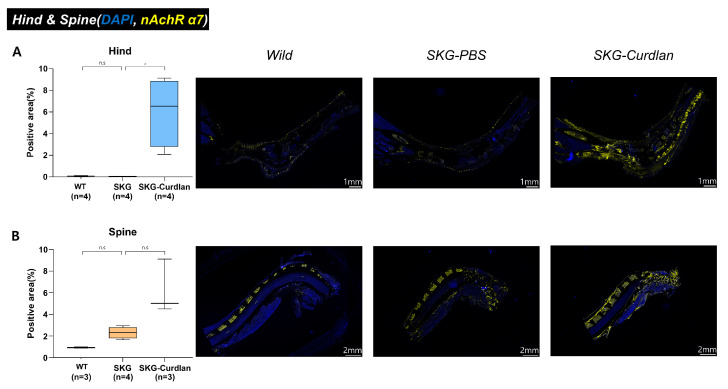
Opal multiplexed immunofluorescence detection of α7 nAChR^+^ and semi-quantitative analysis of positive areas: (**A**) hind tissue and (**B**) spinal tissue. The blue signal is from DAPI, staining the nucleus, and the yellow signal is from α7 nAChR. Mann–Whitney U tests were performed for two-group comparisons, scale bar, 1 mm for hind tissue and 2 mm for spine tissue, ns, not significant, * *p* ≤ 0.05.

First, we performed immunofluorescence staining of the α7 nAChR^+^ cells to assess the levels of α7 nAChR in the curdlan-administered SKG mice. Opal multiplexed immunofluorescent staining was performed on the left hind joint and spine joint. The numbers of α7 nAChR cells were higher in the hind joints and spinal tissue of the curdlan-treated SKG mice than in the wild type mice, but statistical significance was only attained in the hind joints (Figure 1).

### 3.2. Effect of Cigarette Smoke on Peripheral Arthritis

**Figure 2:**
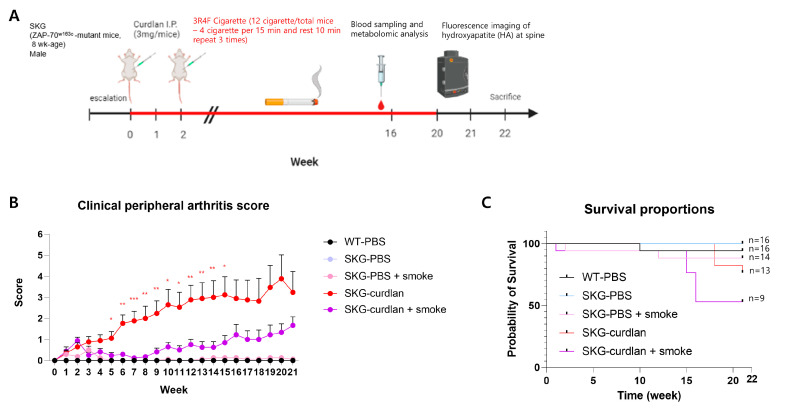
Experimental design and clinical peripheral arthritis scores. (**A**) Experimental design; (**B**) clinical peripheral arthritis scores; (**C**) survival proportions. ANOVA tests were performed for comparison. * *p* ≤ 0.05, ** *p* ≤ 0.01, *** *p* ≤ 0.001.

We evaluated the effect of cigarette smoke in SKG mice according to the schedule illustrated in Figure 2A. From 5 weeks after curdlan injection, worsening of the peripheral arthritis score was significantly less in the smoke-exposed curdlan-administered SKG mice than in the unexposed mice. (Figure 2B) However, this difference was not observed at 16 weeks post curdlan injection because half of the mice exposed to cigarette smoke had died (Figure 2C).

In the metabolic analysis of mouse sera obtained 16 weeks after the first curdlan injection, nicotine was not detected. However, cotinine levels increased significantly in the cigarette-smoke-exposed curdlan-administered SKG mice as validation for smoking exposure (Supplementary Figure S1).

### 3.3. Histologic Examination of Peripheral Joints

**Figure 3:**
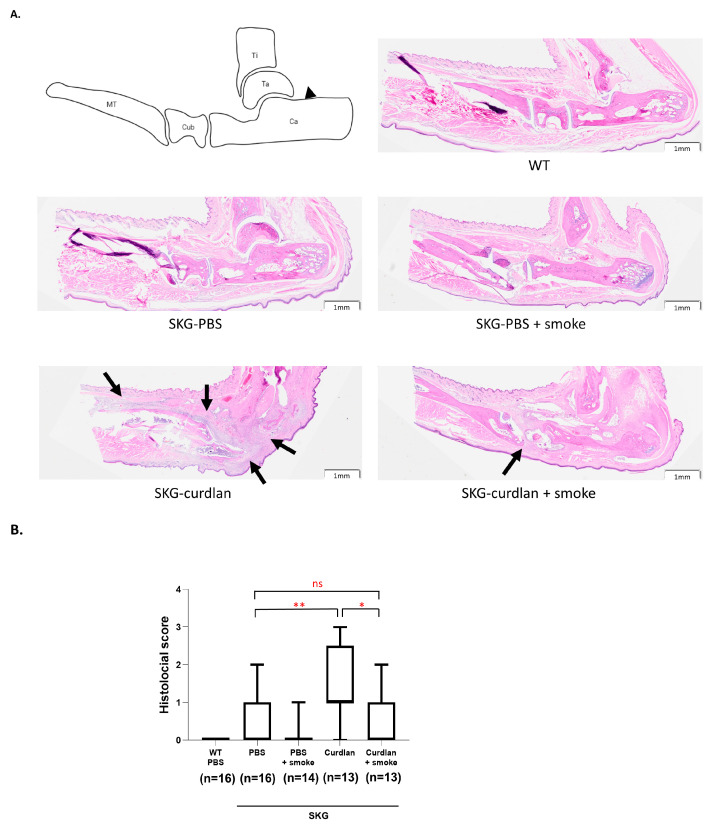
Histology of peripheral joints. (**A**) H&E-stained hind tissue and (**B**) histologic scores of hind joint inflammation. Arrows indicate the infiltrated inflammatory cells, Ca, calcaneus; Cub, cuboid; MT, metatarsal; Ta, talus; Ti, tibia. Mann–Whitney U tests were performed for two-group comparisons. Scale bar, 1 mm. ns, not significant, * *p* ≤ 0.05, ** *p* ≤ 0.01.

For histological examination, the left hind regions of mice were stained with H&E. A schematic anatomical diagram of the hind region, and representative magnified images of the various groups, are shown in Figure 3A. H&E staining revealed joint inflammation with signs of inflammatory cell infiltration (arrows), synovium thickening, and increasing synoviocytes. The curdlan-administered mice that had not been exposed to smoke had the most severe arthritis as indicated by semiquantitative scoring of joint inflammation in accordance with the SMASH. Smoke-exposed curdlan-administered SKG mice showed lesser joint inflammation than mice not exposed (Figure 3B). 

### 3.4. Osteoblast Activity in the Spine

**Figure 4:**
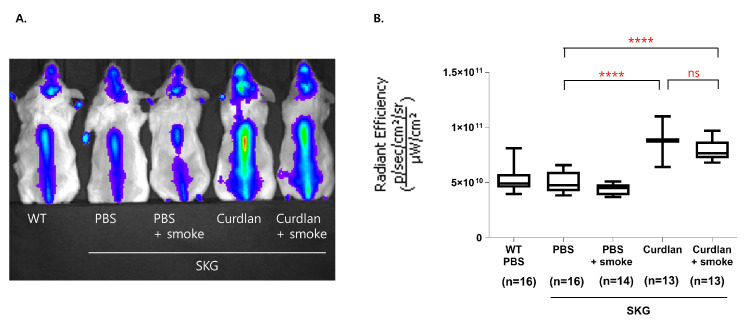
In vivo fluorescence imaging with a bisphosphonate agent. (**A**) Fluorescence imaging of the spine with hydroxyapatite (HA). (**B**) Quantitative analysis of fluorescence. Mann–Whitney U tests were performed for two-group comparisons. ns, not significant, **** *p* ≤ 0.0001.

We also assessed osteoblast activity by evaluating the accumulation of hydroxyapatite using a fluorescent in vivo bisphosphonate imaging agent. A representative distribution of the fluorescence signals in the mouse spine is shown in Figure 4A. The curdlan-administered SKG mice had stronger fluorescence signals than the controls; however, exposure to cigarette smoke did not alter the osteoblast activity in these mice (Figure 4B).

### 3.5. Effect of Cigarette Smoke on the Expression of α7 nAChRs, IL-17A, FOXP3, and F4/80 in the Synovium

**Figure 5:**
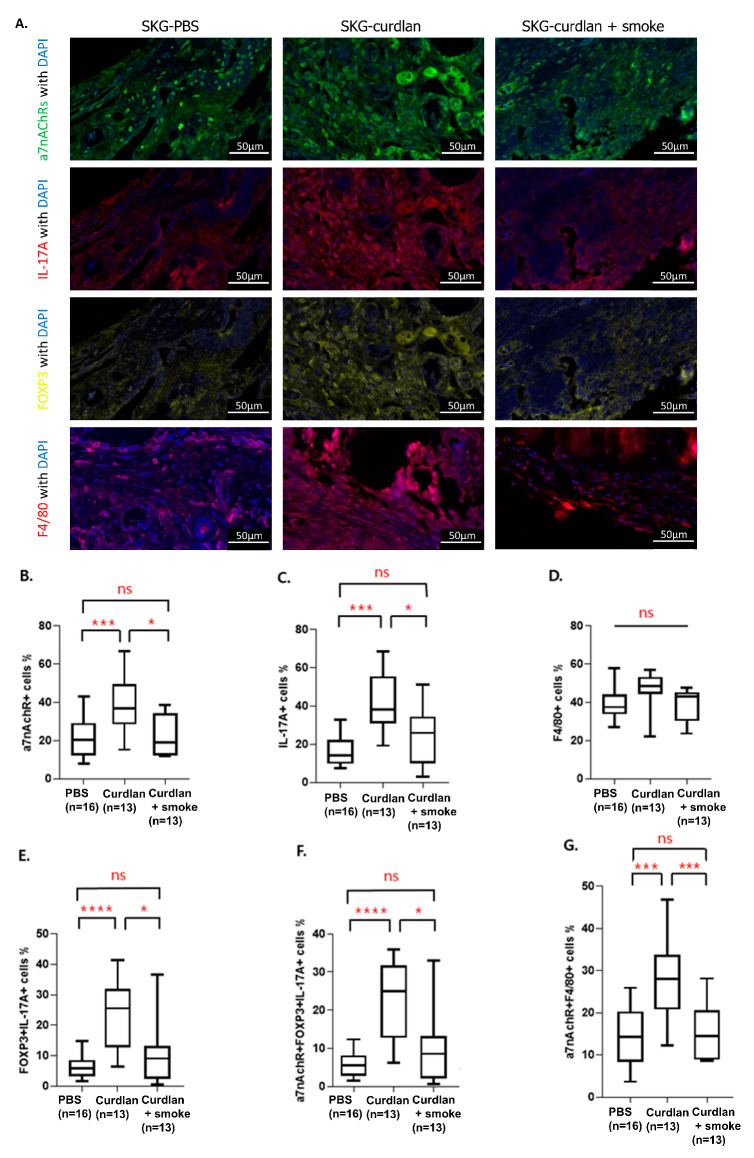
Opal multiplexed immunofluorescence images and semi-quantification. (**A**) Opal multiplexed immunofluorescence images that show α7 nAChRs^+^ cells, IL-17A^+^ cells, FOXP3^+^ cells, and F4/80^+^ cells in the hind tissue. (**B**) α7 nAChRs^+^ cells; (**C**) IL-17A^+^ cells; (**D**) F4/80^+^ cells; (**E**) FOXP3^+^IL17A^+^ cells; (**F**) α7 nAChRs^+^FOXP3^+^IL17A^+^ cells; (**G**) α7 nAChRs^+^F4/80^+^ cells. Mann–Whitney U tests were performed for two-group comparisons. Scale bar, 50 µm. ns, not significant, * *p* ≤ 0.05, *** *p* ≤ 0.001, **** *p* ≤ 0.0001.

We found that numbers of alpha7 nAChRs^+^ cells, α7 nAChRs^+^IL-17A^+^ cells, α7 nAChRs^+^ IL-17A^+^FOXP3^+^ cells, and α7 nAChRs^+^ F4/80^+^ cells were higher in the synovia of curdlan-treated SKG mice than in those of the control SKG mice. However, smoke-exposed curdlan-administered SKG mice had significantly lower levels of the α7 nAChRs^+^ or α7 nAChRs^+^IL-17A^+^ cells than the unexposed curdlan-administered SKG group. The F4/80^+^ cells did not differ significantly between the groups (Figure 5).

## 4. Discussion

Our study aimed to investigate the potentially ameliorative effect of cigarette smoke on curdlan-administered SKG mice, a murine model of autoimmune arthritis with abundant α7 nAChR^+^ cells in the synovia. Curdlan-administered SKG mice had increased numbers of α7 nAChR^+^ and IL-17A^+^ cells and higher clinical arthritis scores in their peripheral joints, and clinical arthritis scores and histologic inflammation were improved in cigarette smoke-exposed curdlan-administered SKG mice, along with a reduction in α7 nAChR^+^ and IL-17A^+^. Our results suggest that exposure to cigarette smoke can influence peripheral arthritis and immune cell populations in curdlan-administered SKG mice. 

SKG mice have characteristics of human SpA, including peripheral arthritis, enthesitis, and inflammatory bowel disease [27]. These mice have a point mutation involving the SH2 domain in the gene encoding ZAP-70, a key signal transduction molecule in T cells [28]. They tend, especially when exposed to a beta-glucan such as a curdlan, to develop peripheral arthritis in the early stage and spinal ankylosis in the late stage due to the development of autoreactive T cells that signal at least in part via IL-17A/IL-22 and IL-23 [29,30]. In line with previous studies, the IL-17A^+^ and FOXP3^+^IL-17A^+^ cells were increased in our curdlan-administered SKG mice. Additionally, the marked expression of α7 nAChR^+^ cells in the synovia, which is detected in the synovia of RA patients, was detected in SKG mice in our study. These findings underscore the relevance of α7 nAChR in the context of SpA.

Cigarette smoking is a poor prognostic factor for RA and AS [25,35]. However, in a study with collagen-induced arthritic mice, cigarette smoke did not significantly aggravate arthritis, and in fact slowed its onset and progression [11]. However, with regard to the relationship between cigarette smoking and the symptoms of SpA, no significant difference was found between current smokers and non-smokers [36]. On the other hand, in an early axial SpA cohort, smoking was independently associated with an earlier onset of inflammatory back pain and increased axial inflammation [37]. Other studies have shown a correlation between back pain or disease activity and smoking, but no correlation between smoking and the time of onset of symptoms [10,25,38,39]. Although there have been several studies on a potential link between habitual smoking and axial SpA [10,25,38,39,40], there is no consistent evidence supporting the independent role of smoking in disease onset. In our study, smoke-exposed mice showed a reduced α7 nAChR expression in the joint tissues, and smoking might have had an antiarthritic role accompanied with a reduction in α7nAChR expression. Thus, this study is especially meaningful as it is the first animal study to demonstrate a relationship between exposure to cigarette smoke and peripheral arthritis in an animal model with characteristics of SpA. While this result offers valuable insights into the immunomodulatory effects of smoking, it is crucial to emphasize that cigarette smoking is associated with a myriad of detrimental health effects and is not a recommended therapeutic approach for arthritis. Instead, our study raises the prospect of exploring nicotine analogs, such as cotinine, which have shown immunomodulatory properties without carrying the same harmful health risks associated with smoking [41,42]. These analogs may hold potential as future therapeutic interventions for managing inflammatory joint conditions while avoiding the adverse consequences of smoking. Further research in this direction is warranted to explore the clinical feasibility and safety of such interventions. 

In SpA, excessive new bone formation occurs in response to mechanical stress, and disease pathogenesis is related to the interaction between inflammatory cells and bone cells [43]. A cohort study of SpA patients suggested that smoking caused structural damage to the spine and induced oxidative stress by increasing superoxide radicals, which inhibited osteogenic differentiation. However, nicotine and cotinine did not directly affect osteogenic differentiation but rather inhibited the osteogenic differentiation of mesenchymal stem cells [44]. In our study, we observed a decrease in the IL-17A^+^ cells in the synovia of smoke-exposed curdlan-administered SKG mice. IL-17A induces enthesitis by promoting osteoclast formation, but its effect on osteoblast differentiation varies depending on the cell type, the differentiation stage of a cell, and the timing and duration of exposure [45]. In our study, cigarette smoke did not alter osteogenic activity in the mice. Osteogenic activity and bone formation might be affected by the oxidative stress induced by cigarette smoke and inflammatory cytokines such as IL-17.

IL-17, which is mainly produced by TH17 cells, plays an important role in inflammatory diseases [46]. The TH17 cells were much more frequent among the peripheral blood monocyte cells of patients with AS than in healthy controls [47]. In the present study, cigarette smoke decreased the proportions of IL-17A^+^, IL-17A^+^FOXP3^+^, and α7 nAChR^+^IL-17A^+^FOXP3^+^ cells in the hind regions of curdlan-administered SKG mice, and led to an anti-arthritogenic response. IL-17A levels may be reduced via a mechanism that inhibits NFκB binding or JAK-STAT signaling using receptor activation [48].

The present study has several limitations. First, the metabolic and histologic analyses were performed at different times. Second, it provides no in vitro insight into the mechanism underlying the association between nicotine and its metabolite cotinine, and peripheral arthritis. In vivo and in vitro experiments with agonists of α7 nAChR are needed to establish how smoke exposure induces SpA-like phenotypes. Further studies are warranted to substantiate the mechanisms underlying smoking’s beneficial effects on arthritis, including a more conclusive elucidation of the specific cell types involved. Additionally, while our research investigated the overall impact of cigarette smoke exposure on α7 nAChR-expressing immune cells and the associated immune response, we neither isolated nor analyzed the effects of nicotine or of the α7 nAChR agonist alone. Tobacco smoke contains various substances. Especially nitrosamines such as nitrosamines 4-(methylnitrosamino)-1-(3-pyridyl)-1-butanone (NNK) and N′-nitrosonornicotine (NNN) have immunomodulatory effects, mostly in tumor pathogenesis [49]; therefore, further study is warranted. Finally, the notable increase in mortality among SKG-curdlan mice after prolonged smoke exposure requires further explanation. Smoke exposure, particularly over an extended period, can have diverse physiological effects that are beyond the scope of our study.

In conclusion, we showed that an increase in the α7 nAChR^+^ cells within the joint tissue of curdlan-administered SKG mice and smoke exposure following curdlan administration appears to influence peripheral arthritis and immune cell populations in curdlan-administered SKG mice. 

## Data Availability

The data underlying this article will be shared on reasonable request by the corresponding author.

## Supplementary Materials

The following supporting information can be downloaded at https://www.mdpi.com/article/10.3390/biomedicines11102757/s1, Figure S1: Cotinine levels in mice serum.
