# Supplementary material for: The Expression of the Alpha7 Nicotinic Acetylcholine Receptor and the Effect of Smoking in Curdlan-Administered SKG Mice
Source: Biomedicines. 2023 Oct 11;11(10):2757. doi: 10.3390/biomedicines11102757 (PMC10603960; doi:10.3390/biomedicines11102757)
Supplement: Supplementary file 1 [file biomedicines-11-02757-s001.zip › biomedicines-2627585-supplementary.pdf]

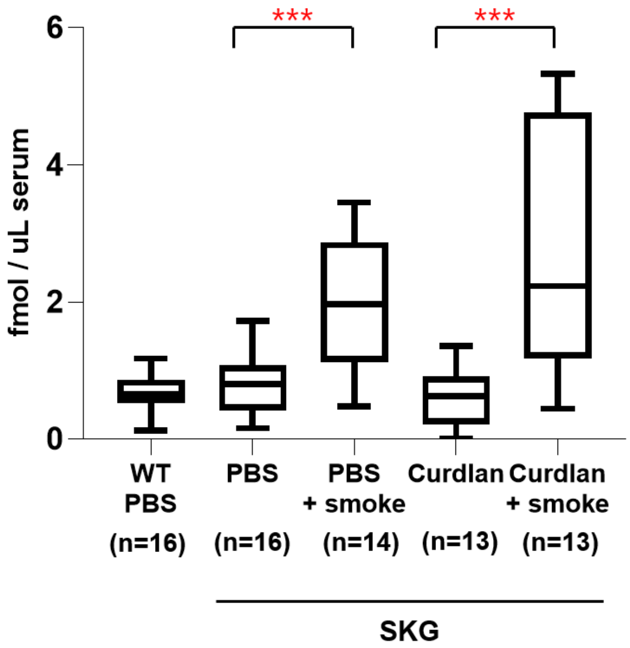

Figure S1. Serum cotinine level of mice serum. Mann–Whitney U tests were performed for two-group comparisons. ***$p \leq 0.001$.
